# Elevated Expression of the Long Noncoding RNA MAFTRR in Patients with Hashimoto's Thyroiditis

**DOI:** 10.1155/2021/3577011

**Published:** 2021-11-26

**Authors:** Huiyong Peng, Xiangmei Ding, Juan Xu, Yue Han, Jun Yang, Xinyi Tang, Shengjun Wang, Yingzhao Liu

**Affiliations:** ^1^Department of Laboratory Medicine, The Affiliated People's Hospital of Jiangsu University, Zhenjiang Medical School of Nanjing Medical University, Zhenjiang 212002, China; ^2^Department of Genetic Toxicology, The Key Laboratory of Modern Toxicology of Ministry of Education, Center for Global Health, School of Public Health, Nanjing Medical University, Nanjing 211100, China; ^3^Department of Endocrinology, The Affiliated People's Hospital of Jiangsu University, Zhenjiang Medical School of Nanjing Medical University, Zhenjiang 212002, China; ^4^Department of Critical Care Medicine, The Affiliated People's Hospital of Jiangsu University, Zhenjiang Medical School of Nanjing Medical University, Zhenjiang 212002, China; ^5^Division of Hematology and Internal Medicine, Mayo Clinic, Rochester, MN 55902, USA

## Abstract

**Background:**

Long noncoding RNAs (lncRNAs) represent an important novel class of noncoding RNA molecule greater than 200 nucleotides that play a key role in the regulation of autoimmune diseases. Previous studies have demonstrated that MAFTRR (MAF transcriptional regulator RNA) regulated Th1 cells differentiation by inhibiting the expression of MAF in activated CD4^+^ T cells. However, the effect of MAFTRR on the pathogenesis of Hashimoto's thyroiditis (HT) remains unclear. This research was aimed at investigating the expression of MAFTRR in Hashimoto's thyroiditis (HT) as well as the correlation between MAFTRR and Th1 cells.

**Methods:**

Thirty-eight HT patients and thirty-eight healthy controls were enrolled in the study. The proportion of Th1 cells and CD8^+^IFN-*γ*^+^ T cells in peripheral blood mononuclear cells (PBMCs) from these specimens was determined by flow cytometric analysis. The transcript levels of MAFTRR, MAF, and IFNG in PBMCs and thyroid glands were detected by quantitative real-time PCR. Receiver operating characteristic (ROC) curve analysis was performed to evaluate the potential value of MAFTRR in the HT patients.

**Results:**

We found that the proportion of circulating Th1 cells and the transcript levels of IFNG were increased in peripheral blood of the HT patients. The transcript levels of MAFTRR were significantly increased in the HT patients and positively correlated with the percentage of Th1 cells and serum levels of antithyroglobulin antibody and antithyroperoxidase antibody. The transcript levels of MAF, a transcription factor that inhibits Th1 cells activity and IFN-*γ* production, were attenuated in PBMCs from the HT patients. The transcript levels of IFNG had positive and inverse correlations with MAFTRR and MAF expression in PBMCs from the HT patients, respectively. Additionally, a significantly positive correlation between upregulated MAFTRR expression and augmented IFNG expression was revealed in thyroid tissues from the HT patients. ROC curve suggested that MAFTRR could potentially differentiate the HT patients from healthy controls.

**Conclusion:**

MAFTRR is significantly augmented in the HT patients and may contribute to the pathogenic role of the Th1 cells response in HT.

## 1. Introduction

Hashimoto's thyroiditis (HT) is a chronic inflammatory disorder of the thyroid gland [1]. It was first described in Japan by Dr. Hakaru Hashimoto over a century ago [2]. The clinical features of HT patients are characterized by increased thyroid volume, lymphocyte infiltration of interstitium between thyroid follicles, and the presence of specific antibodies to thyroid antigens, including antithyroglobulin antibody (TgAb) and antithyroperoxidase antibody (TPOAb) [3, 4]. HT was long considered a rare disease until the change in diagnostic modality, mostly, the introduction of thyroid function and thyroid antibodies tests [5]. It is now considered the most common autoimmune disease and the most common endocrine disorder [6]. Studies have demonstrated that the development of HT depends on an immune deficiency caused by individual genetic susceptibility together with environmental factors [7, 8]. However, the pathogenesis of HT has not been fully comprehended.

Long noncoding RNAs (lncRNAs), one of the family members of noncoding RNAs, are recently appreciated as key regulatory layers of genome expression [9]. They are highly prevalent in the eukaryotic transcriptome, and composed of >200 nucleotides, shorter in length (relative to the mRNA), contained fewer albeit longer exons, low evolutionary sequence conservation, and expressed at lower levels in comparison with mRNAs [10]. Research evidence showed that many abnormal lncRNAs, such as lnc-DC, lincRNA-Cox2, and Tmevpg1, are involve in the immune system [11–13]. The lncRNA transcript MAFTRR (MAF transcriptional regulator RNA), also known as linc-MAF-4, was a chromatin-associated Th1-specific lncRNA [14]. MAFTRR regulated Th1 cells differentiation by inhibiting the expression of MAF in activated CD4^+^ T cells [15]. Maf was a Th2-associated transcription factor, which was poorly expressed in Th1 cells and decreased the production of interferon *γ* (IFN-*γ*) [16]. Studies focused on immune cells have indicated that Th1 cells play an important role in HT [4, 17], but the molecular mechanisms causing Th1 cells imbalance remain unknown. The relationship between MAFTRR and Th1 cells inspired us to hypothesize that MAFTRR may play a crucial pathogenic role in the development of HT.

Herein, we investigated whether MAFTRR was dysregulated in the HT patients and analyzed the correlation between MAFTRR and Th1 cells. Using this approach, we hope to elucidate the role and the potential value of MAFTRR in HT.

## 2. Materials and Methods

### 2.1. Subjects and Samples

HT specimens were collected in the Department of Clinical Laboratory of the Affiliated People's Hospital of Jiangsu University. Thirty-eight adult HT patients (32 females and 6 males) were enrolled into the study. HT patients were diagnosed by diffuse goiter, increased serum concentrations of TgAb and/or TPOAb, and heterogeneous echotexture in those who accepted B-ultrasonic examination. Fifteen HT patients with hypothyroidism had increased levels of thyroid stimulating hormone (TSH), or combined with low levels of free triiodothyronine (FT3) and/or free thyroxine (FT4). Other HT patients were euthyroid. The criteria of healthy control group were as follows: (i) normal thyroid function and thyroid B-ultrasound; (ii) no occurrence of recent infection; (iii) no autoimmune diseases and no recently taken immunosuppressive drugs; and (iv) the age matched with HT group. Thirty-eight age- and sex-matched healthy adult subjects were collected in the Physical Examination Center of the Affiliated People's Hospital of Jiangsu University as the controls. All healthy subjects were euthyroid and free of thyroid-specific autoantibodies. Healthy participants with autoimmune diseases, tumors, and infectious diseases were excluded from the present study. The numbers of peripheral leukocytes in all individuals were within the normal range (3.50 − 9.50 × 10^9^/l). All subjects were not taking immunosuppressive drugs or drugs that affect thyroid function. Peripheral blood samples were obtained from the HT patients and healthy controls. Fresh tissue samples from the thyroid gland of seven HT patients were collected and stored at -80°C until use. Fresh thyroid tissues from nine patients with simple goiter were used as control. All tissue samples were confirmed by clinicopathological diagnosis.

All protocols were approved by the Ethics Committee of The Affiliated People's Hospital of Jiangsu University (K-20180009-Y), and the samples were collected after obtaining the written informed consent of all subjects. All procedures described in this study were compliant with the standard biosecurity and institutional safety procedures.

### 2.2. Laboratory Measurements

Serum from participants was used to measure indicators of thyroid function. The serum levels of FT3 (3.28-6.47 pmol/L), FT4 (7.64-16.03 pmol/L), TSH (0.56-5.91 uIU/ml), TgAb (0-4 IU/ml), and TPOAb (0-9 IU/ml) were measured by chemiluminescent immunoassay using an LDX-800 system (Beckman Coulter, CA, USA) according to the manufacturer's instructions.

### 2.3. Cell Isolation and Purification

Human peripheral blood mononuclear cells (PBMCs) were separated from fresh anticoagulated blood by Ficoll-Hypaque density gradient centrifugation (Haoyang Biological Technology Co., Tianjin, China) until use for flow cytometric analysis [18] and quantitative real-time PCR (qRT-PCR). Human PBMCs were cultured in RPMI-1640 medium (Gibco, California, USA) supplemented with 10% fetal bovine serum (Gibco) for further study.

### 2.4. Flow Cytometric Analysis

For intracellular cytokine assays, PBMCs were resuspended at 1 × 10^6^/ml in RPMI-1640 medium containing 10% fetal bovine serum and stimulated with 50 ng/ml of phorbol myristate acetate (PMA; Sigma-Aldrich, California, USA) and 1 *μ*g/ml of ionomycin (Sigma-Aldrich) for 2 hours and then incubated for an additional 4 hours in the presence of 1 *μ*g/ml of brefeldin-A (eBioscience, San Diego, USA) at 37°C in 5% CO_2_. Then, the suspended cells were stained with phycoerythrin-cyanin 5- (PE-Cy5-) conjugated anti-human CD3 mAb and fluorescein isothiocyanate- (FITC-) conjugated anti-human CD8 mAb (eBioscience) against cell surface antigens for 30 minutes at 4°C in the dark. Then, the cells were fixed and permeabilized using an intracellular staining kit (Invitrogen, Carlsbad, USA), followed by incubation for 45 minutes at 4°C in the dark with PE-conjugated anti-human IFN-*γ* mAb (Miltenyi Biotec GmbH, Bergisch Gladbach, Germany). The data were analyzed using FlowJo 10 (Stanford University, San Francisco, USA). In this study, CD3^+^CD8^−^IFN-*γ*^+^ cells and CD3^+^CD8^+^IFN-*γ*^+^ cells were defined as Th1 cells and CD8^+^IFN-*γ*^+^ T cells, respectively.

### 2.5. RNA Extraction and qRT-PCR

Total RNA was isolated from PBMCs with TRIzol reagent (Invitrogen, California, USA) according to the manufacturer's instructions. Reverse transcription (Toyobo, Osaka, Japan) was performed to synthesize cDNA according to the manufacturer's instructions using the random and oligo-dT primers provided in the ReverTraAca® qPCR RT kit (Toyobo). qRT-PCR was performed using TaKaRa TB Green™ Premix Ex Taq II (TaKaRa, Osaka, Japan). The primer sequences used for qRT-PCR were as follows: MAFTRR, sense, 5′-TACCTGCTAGGCTCGTCCCA-3′, antisense, 5′-TAGAAGGAGGCACCCGCTTG-3′; MAF, sense, 5′-TGGCAATGAGCAACTCCGAC-3′, antisense, 5′-CACTGGCTGATGATGCGGTC-3′; IFNG, sense, 5′-GAGTGTGGAGACCATCAAGGA-3′, antisense, 5′-TGTATTGCTTTGCGTTGGAC-3′; and *β*-actin, sense, 5′-CACGAAACTACCTTCAACTCC-3′, antisense, 5′-CATACTCCTGCTTGCTGATC-3′. The data were analyzed using the Applied Biosystems 7500 Manager software v2.3 (Thermo Fisher Scientific, Waltham, MA, USA).

### 2.6. Statistical Analysis

The GraphPad Prism version 5 software (GraphPad Software, Inc., San Diego, USA) was use to manage and analyze the data. A Student's unpaired *t*-test was used for comparisons of two groups when variables passed the normal distribution test. The Mann–Whitney *U* test was performed to analyze the differences between the two groups of nonnormally distributed data. Correlation between the variables was determined by the Pearson's correlation coefficient. Receiver operating characteristic (ROC) curve and the area under ROC curve (AUC) were generated based on the logistic regression model. Sensitivity was used as the *y*-axis to represent true positive rate, while 100% − specificity% was used as the *x*-axis to represent false positive rate. All experiments were performed three times. *p* < 0.05 was considered to indicate a statistically significant difference. (^∗^*p* < 0.05, ^∗∗^*p* < 0.01, ^∗∗∗^*p* < 0.001).

## 3. Results

### 3.1. The Clinical Characteristics of the Subjects in the Study

Thirty-eight HT patients and 38 healthy controls were enrolled in the study. The age of HT group and control group was 45 ± 13 and 43 ± 12, respectively. The male-to-female ratio of HT patients is about 1 : 5.3. Among them, 15 HT patients with hypothyroidism had increased serum levels of TSH, and two patients combined with low levels of FT3 and FT4. Other HT patients were euthyroid. All patients had elevated concentrations of TPOAb, and the serum levels of TgAb were also augmented in 36 patients. The serum levels of FT3, FT4, TSH, TgAb, and TPOAb in all healthy individuals were within the normal range (Table 1).

### 3.2. Increased Expression of MAFTRR in PBMCs from the HT Patients

**Figure 1:**
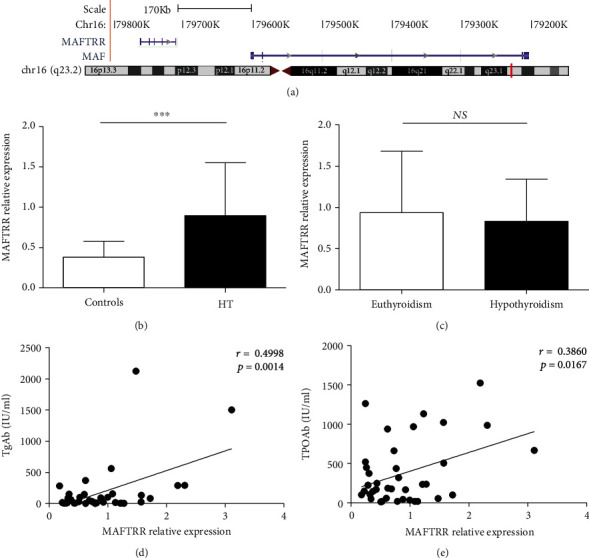
Increased expression of MAFTRR in PBMCs from the HT patients. (a) Genomic position of MAFTRR and MAF on human chromosome. (b) The transcript levels of MAFTRR in PBMCs from 38 HT patients and 38 healthy controls were determined by qRT-PCR. (c) The transcript levels of MAFTRR in PBMCs from the HT patients with euthyroidism and hypothyroidism were determined. The correlations between the transcript levels of MAFTRR and the serum concentrations of TgAb (d) and TPOAb (e) in the HT patients are shown. Each data point represents an individual subject, and the horizontal lines show the mean. ^∗∗∗^*p* < 0.001. *NS*: no significance.

MAFTRR is a long noncoding RNA comprised of six exons and located at Chromosome 16q23 (Figure 1(a)). To investigate MAFTRR expression in the HT patients, PBMCs from participants were used to detect the transcript levels of MAFTRR by qRT-PCR. As shown in Figure 1(b), the relative expression of MAFTRR from PBMCs was significantly higher than that in healthy controls. Then, we analyzed MAFTRR expression in the HT patients with euthyroidism and hypothyroidism, and the result showed that there was no difference in MATRR expression among them (Figure 1(c)). As shown by correlation analysis, MAFTRR expression was positively correlated with the levels of TgAb (*r* = 0.4998, *p* = 0.0014) (Figure 1(d)) and TPOAb (*r* = 0.3860, *p* = 0.0167), respectively (Figure 1(e). These results showed that the levels of MAFTRR were increased in the HT patients and associated with the process of HT. However, this indicator did not distinguish between clinical subtypes of HT.

### 3.3. The Comparison of MAF Expression between the HT Patients and Healthy Controls

**Figure 2:**
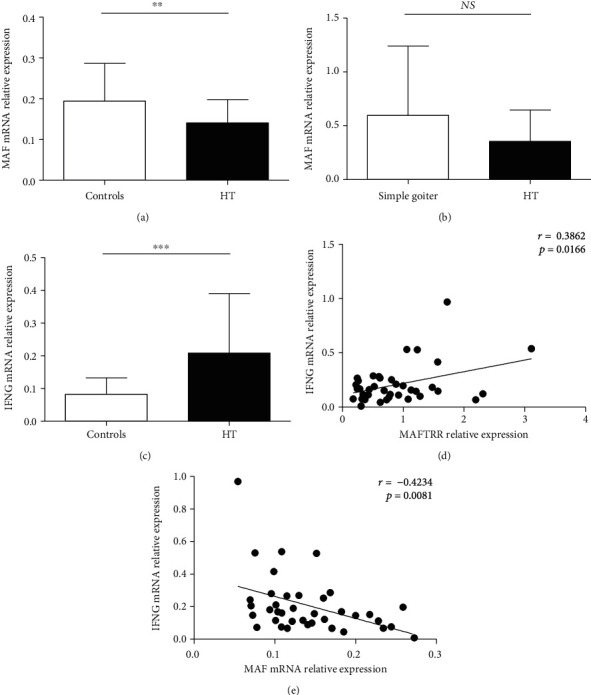
The comparison of MAF expression between the HT patients and healthy controls. (a) The relative expression of MAF mRNA in PBMCs from the HT patients and healthy controls was detected by qRT-PCR. (b) The relative expression of MAF mRNA in the thyroid glands of the HT patients and healthy controls was determined. (c) The relative expression of IFNG mRNA was determined in PBMCs from the HT patients and healthy controls. (d) The correlation between the transcript levels of MAFTRR and the transcript levels of IFNG in PBMCs from the HT patients is shown. (e) The correlation between the transcript levels of MAF and the transcript levels of IFNG in PBMCs from the HT patients was shown. Each data point represents an individual subject, and the horizontal lines show the mean. ^∗∗^*p* < 0.01, ^∗∗∗^*p* < 0.001. *NS*: no significance.

MAFTRR transcript is positioned approximately 170 kb from the MAF coding region (Figure 1(a)). MAFTRR is the cellular inhibitor of MAF, and we detected the expression of MAF in PBMCs from the HT patients and healthy control subjects using qRT-PCR. As shown in Figure 2(a), the transcript levels of MAF in PBMCs were decreased in the HT patients compared with healthy subjects. As the downstream regulatory gene of MAF, our data also revealed that the transcript levels of IFNG were significantly upregulated in PBMCs from the HT patients (Figure 2(c)). Notably, we found that IFNG expression was positively correlated with MAFTRR expression (*r* = 0.3862; *p* = 0.0166) (Figure 2(d)), and a notable inverse correlation between IFNG transcripts levels and MAF transcripts levels was also found in PBMCs from the HT patients (*r* = −0.4234; *p* = 0.0081) (Figure 2(e)). These results suggested that MAFTRR, MAF, and IFNG were closely related in HT, and their expression in peripheral blood from HT was consistent with the regulating relationship. However, the levels of MAF, by contrast were not changed in the thyroid tissues from the HT patients compared with that from the simple goiter patients (Figure 2(b)). The inconsistent expression of MAF in peripheral blood and thyroid tissues might be due to the small size of tissue samples. Concordant with these findings, we hypothesized that elevated expression of MAFTRR in peripheral blood from the HT patients might inhibit MAF transcription and further upregulate IFNG expression.

### 3.4. Positive Correlation between MAFTRR Expression and Circulating Th1 Cells in the HT Patients

**Figure 3:**
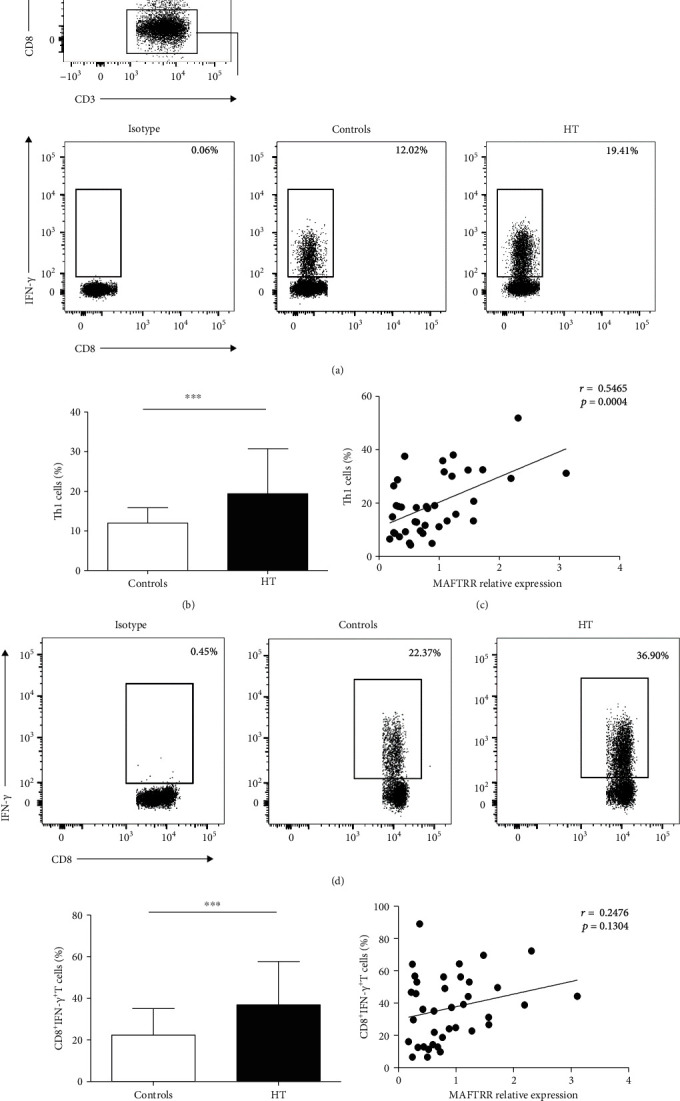
Positive correlation between MAFTRR expression and circulating Th1 cells in the HT patients. Fresh peripheral blood was collected, and the PBMCs were subsequently separated from peripheral blood within four hours and analyzed the proportion of Th1 cells by flow cytometry analysis. (a) Representative flow cytometry dot plots of Th1 cells are indicated. Values in the upper left rectangular region correspond to the proportion of Th1 cells. (b) The proportion of Th1 cells is shown in the peripheral blood from the HT patients and controls. (c) The correlation between the transcript levels of MAFTRR and the percentages of Th1 cells is shown in the peripheral blood from the HT patients. (d) Values in the upper right rectangular region correspond to the proportion of CD8^+^IFN-*γ*^+^ T cells. (e) The percentages of CD8^+^IFN-*γ*^+^ T cells are indicated in the peripheral blood from the HT patients and controls. (f) The correlation between the transcript levels of MAFTRR and the percentages of CD8^+^IFN-*γ*^+^ T cells is shown in the peripheral blood from the HT patients. Each data point represents an individual subject, and the horizontal lines show the mean. ^∗∗∗^*p* < 0.001.

To address the possibility that MAFTRR contributes to Th1 cells in the HT patients, the proportion of Th1 cells was detected by flow cytometric analysis. We analyzed the proportion of CD3^+^CD8^−^IFN-*γ*^+^ cells in PBMCs to distinguish Th1 cells (Figure 3(a)). The proportion of Th1 cells were significantly increased in PBMCs from the HT patients (Figure 3(b)). Moreover, a significantly positive correlation was observed between MAFTRR expression and the percentage of Th1 cells (*r* = 0.5465; *p* = 0.0004) (Figure 3(c)). We also found that the percentage of CD8^+^IFN-*γ*^+^ T cells (Figure 3(d)) was increased in PBMCs from the HT patients compared with healthy controls (Figure 3(e)). However, there was no correlation between the relative expression of MAFTRR and the percentage of CD8^+^IFN-*γ*^+^ T cells (*r* = 0.2476; *p* = 0.1304) (Figure 3(f)). These findings collectively suggested that the high levels of MAFTRR evoked the proportion of Th1 cells in the HT patients, but not the IFN-*γ* produced by CD8^+^ T cells.

### 3.5. Elevated Levels of MAFTRR and IFNG in Thyroid Tissues from the HT Patients

**Figure 4:**
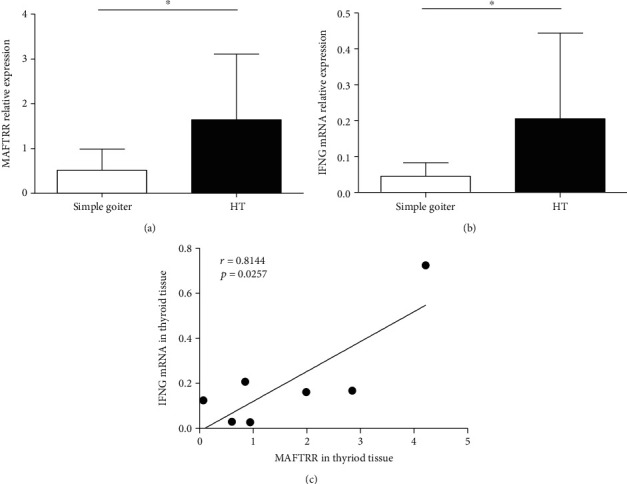
Elevated levels of MAFTRR and IFNG in thyroid tissues from the HT patients. (a) The transcript levels of MAFTRR in thyroid glands of patients with HT and patients with simple goiter were determined by qRT-PCR. (b) The transcript levels of IFNG in thyroid glands of patients with HT and patients with simple goiter were determined by qRT-PCR. (c) The correlation between the transcript levels of MAFTRR and the transcript levels of IFNG in thyroid glands of the HT patients is shown. Each data point represents an individual subject, and the horizontal lines show the mean. ^∗^*p* < 0.05.

HT is an organ-specific autoimmune disease characterized by lymphoid infiltration. To investigate the expression of MAFTRR in thyroid tissues, fresh thyroid tissues were collected to detect the transcript levels of MAFTRR and IFNG. Results showed that the relative expression of MAFTRR (Figure 4(a)) and IFNG (Figure 4(b)) in the thyroid glands from the HT patients were higher than that from patients with simple goiter. Moreover, a strong positive correlation between the transcript levels of MAFTRR and IFNG (*r* = 0.8144, *p* = 0.0257) was observed in thyroid glands from the HT patients (Figure 4(c)). These data indicated the consistency of MAFTRR and IFNG expression in the peripheral blood and thyroid tissue.

### 3.6. Potential Diagnostic Value of MAFTRR in HT

**Figure 5:**
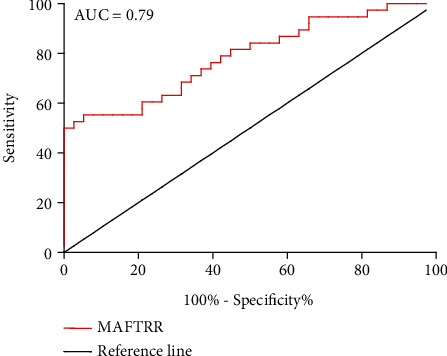
Potential diagnostic value of MAFTRR in HT. The ROC curve analysis of MAFTRR was performed to distinguish the HT patients from the healthy volunteers.

The double-blind method was used in the experiments. The ROC curve analysis was performed to evaluate the potential value of MAFTRR in PBMCs from the HT patients. The AUC of MAFTRR was 0.79 (95%confidence interval (CI) = 0.69 − 0.89, *p* < 0.001), and the sensitivity, specificity, likelihood ratio, and Jorden index were 55.26% and 94.74%, 10.50, and 0.50, respectively (Figure 5). These data showed that MAFTRR could differentiate the HT patients from healthy controls, which might be used as a potentially biomarker for HT.

## 4. Discussion

The Th1 subset and related cytokine IFN-*γ* are implicated in the pathogenesis of HT [19, 20]. Th1 cells are the predominant lymphocytes in the thyroid tissues from the HT patients which directly destroy thyroid follicular cells by activating cytotoxic lymphocytes and macrophages [7]. The previous studies have indicated that the proportion of peripheral Th1 cells was significantly increased in patients with HT [4, 21]. Therefore, the factors contributing to Th1 cells in HT deserve further investigation. MAFTRR was an important regulator of Th1/Th2 polarization. Upregulation of MAFTRR resulted in the skewing of CD4^+^ T cells differentiation towards Th1 cells, while downregulation of MAFTRR, by contrast, heightened the development of Th2 cells in vitro [15]. This means that MAFTRR is a necessary element for Th1 cell differentiation. In addition, MAFTRR can promote IFN-*γ* production in Th1 cells [15]. However, little is known regarding the role of MAFTRR in the pathogenesis of HT. Based on this, overexpression of MAFTRR were revealed in PBMCs from the HT patients. We then gated on CD3^+^CD8^−^ cells as CD4^+^ T cells and identified IFN-*γ*^+^ cells to distinguish Th1 cells owing to CD4 molecule on PBMCs decreased dramatically upon PMA stimulation [22]. Our data showed that the proportion of Th1 cells and the transcript levels of IFNG were increased in PBMCs from patients with HT. Moreover, positive correlations between MAFTRR transcript levels and increased proportion of Th1 cells and IFNG transcript levels were found in peripheral blood from the HT patients. Our results, combined with literature reports, suggested that increased circulating Th1 cells were associated with elevated levels of MAFTRR in HT. Previous studies indicated that overexpression of Maf in tumor specific CD8^+^ T cells also leads to a strong repression of IFN-*γ* production [23, 24]. However, there was no correlation between MAFTRR expression and the percentage of effector CD8^+^ T cells, which also produced a significant amount of IFN-*γ* in the HT patients. One possible interpretation is the different role of MAFTRR between Th1 and CD8^+^ T cells. Another possible interpretation is the involvement of different Maf activities in tumors and autoimmune diseases. The underlying mechanisms remain to be further elucidated. As a typical organ-specific autoimmune disease, we further investigated the levels of MAFTRR and IFNG in the thyroid tissues. As expected, the transcript levels of MAFTRR and IFNG were substantially augmented in the thyroid gland from the HT patients. Additionally, a significantly positive correlation between the transcript levels of MAFTRR and IFNG was found in thyroid tissues from the HT patients. These data indicated that MAFTRR expression was consistently expressed in peripheral blood and thyroid tissues. TgAb and TPOAb are the generally critical diagnostic indicators of HT, and their levels can, to a certain extent, reflect the severity of the disease [25]. We assessed the relationship between MAFTRR and the clinical parameters of HT, and the results indicated that there were positive correlations between the levels of MAFTRR and the concentrations of TgAb and TPOAb in the HT patients. These data suggested that MAFTRR expression, to some extent, mirrored the disease severity of HT. Based on our findings, elevated MAFTRR expression might contribute to Th1 cells response in the HT patients.

Several studies have shown that lncRNAs promote the expression of neighboring genes in a manner dependent on epigenetic modification [26, 27]. In examples as HOTAIR and NeST, HOTAIR severed as a scaffold to induce PRC2 to HOXD gene loci to suppresses the transcription of HOXD by catalyzing H3K27me3 [28]. NeST induced active transcription of IFNG by binding to WDR5, a component of the H3K4 methyltransferase complex [29]. MAFTRR gene maps in relative proximity to MAF gene on chromosomes, which has been found to negatively regulate MAF expression [14]. The mechanism was that MAFTRR might act as a scaffold to recruit two repressors (EZH2 and LSD1) to the promoter of MAF gene, thus hindering its transcription [14]. To better understand the potential role of MAFTRR expression in the HT patients, the transcript levels of MAF were determined. As expected, the transcript levels of MAF from PBMCs were decreased in the HT patients compared with that in healthy controls. In thyroid tissues, there was a modest decreasing trend of MAF expression in the HT patients, which might be due to insufficient sample size. These results suggested that the attenuated MAF might be caused by the augmented MAFTRR in the HT patients. However, there was no direct evidence that MAFTRR regulated MAF in Th1 cells. Although MAFTRR knockdown led to increased MAF expression in activated naïve CD4^+^ T cells, and Th1 cells had low expression of MAF and abundant MAFTRR transcripts [14], these could only indirectly indicate that high expression of MAFTRR correlated with a low amount of MAF transcript in Th1 cells. Further studies will be necessary to asses that MAFTRR regulates MAF in Th1 cells.

The MAF gene, which belonged to AP-1 superfamily, functions as an indispensable regulator in the differentiation of Th1 cells [30, 31]. Studies have shown that Maf, and its human ortholog, MAF, are decreased in mouse and human Th1 cells, respectively [14, 32]. Ectopic expression of Maf in mature Th1 cells granted them the ability to decrease their production of IFN-*γ*, thus dampening their pathogenic immune response [16, 33]. To determine the effect of MAF on IFNG allows us to better understand the role of MAFTRR. In this study, IFNG expression inversely correlated with MAF expression in PBMCs from the HT patients, which indicated that MAFTRR may indirectly affect IFNG expression in peripheral blood through enervating MAF, thus participating in the occurrence of HT. However, the detailed mechanisms of MAFTRR regulating Th1 cells remain unclear and require further investigation.

TgAb and TPOAb are the important diagnostic indicators of HT. However, these antibodies are not specific in the diagnosis of thyroid disease types, which also exist in the serum of some Graves' disease patients [34, 35]. We aimed to identify new biomarkers for HT. Hence, we assessed the efficiency of MAFTRR as a biomarker for HT. Our results indicated that the AUC of MAFTRR in PBMCs of the HT patients was up to 0.79, and the sensitivity, specificity, likelihood ratio, and Jorden index were 55.26%, 94.74%, 10.50, and 0.50, respectively. The results indicated that MAFTRR might be used as a potential biomarker. The results of this study reveal an interesting phenomenon and provide clues for further research on the diagnostic value of lncRNAs in HT. However, this study had some limitations. For instance, the serial study of MAFTRR and HT in Chinese Han Population and our conclusions do not reflect all ethnic groups. Furthermore, the mechanisms of MAF impairing IFN-*γ* in Th1 cells have not been fully elucidated. Finally, the mechanisms that causing elevated MAFTRR levels in HT remain unknown. The posttranscriptional regulation of the transcriptome is a widely studied process that is involved in the epigenetic effects on gene expression [36]. Therefore, whether the increased expression of MAFTRR in HT is involved in epigenetic regulation deserves further study. Taken together, our findings demonstrate that MAFTRR is significantly augmented in the HT patients and may contribute to the pathogenic role of Th1 cells response in the HT patients, although additional studies are needed.

## Figures and Tables

**Table 1:** Clinical features of HT patients and healthy controls induced in the study.

	HT patients	Healthy controls	Range	*p* values
Number	38	38		
Gender (M/F)	6/32	8/30		0.56
Age (year)	45 ± 13	43 ± 12		0.51
FT3 (pmol/liter)	4.87 ± 0.83	5.34 ± 0.59	3.28-6.47	0.01
FT4 (pmol/liter)	10.68 ± 2.35	10.89 ± 1.48	7.64-16.03	0.65
TSH (uIU/ml)	9.08 ± 16.66	2.13 ± 0.90	0.56-5.91	0.01
TgAb (IU/ml)	179.49 ± 414.93	0.38 ± 0.69	0-4	<0.01
TPOAb (IU/ml)	377.65 ± 405.89	1.15 ± 0.90	0-9	<0.01

Data correspond to the arithmetic mean ± SD and were compared using unpaired *t*-tests. M: male; F: female.

## Data Availability

The data used to support the findings of this study are included within the article.

## Additional Points

*Abbreviated Title*. Elevated expression of MAFTRR in HT.
